# A descriptive study of the clinical impacts on COVID-19 survivors using telemonitoring (The TeleCOVID Study)

**DOI:** 10.3389/fmedt.2023.1126258

**Published:** 2023-03-20

**Authors:** Josephine Sau Fan Chow, Annamarie D’Souza, Megan Ford, Sonia Marshall, Susana San Miguel, Ahilan Parameswaran, Mark Parsons, Jacqueline Ramirez, Rumbidzai Teramayi, Nutan Maurya

**Affiliations:** ^1^ Clinical Innovation & Business Unit, South Western Sydney Local Health District, Sydney, NSW, Australia; ^2^ South Western Sydney Nursing and Midwifery Research Alliance, Ingham Institute for Applied Medical Research, Sydney, NSW, Australia; ^3^ Faculty of Medicine, University of New South Wales, Sydney, NSW, Australia; ^4^ Faculty of Medicine, Western Sydney University, Sydney, NSW, Australia; ^5^ Research Directorate, South Western Sydney Local Health District, Liverpool, NSW, Australia; ^6^ Clinical Trial Support Unit, Ingham Institute for Applied Medical Research, Liverpool, NSW, Australia; ^7^ District Nursing and Midwifery Office, South Western Sydney Local Health District, Liverpool, NSW, Australia; ^8^ Emergency Department, Bankstown Hospital, South Western Sydney Local Health District, Liverpool, NSW, Australia; ^9^ Neurology Research Unit, South Western Sydney Local Health District, Liverpool, NSW, Australia

**Keywords:** COVID survivors, long COVID, post-COVID clinical symptom, virtual monitoring, model of care

## Abstract

**Background:**

There is increasing evidence that COVID-19 survivors are at increased risk of experiencing a wide range of cardiovascular complications post infection; however, there are no validated models or clear guidelines for remotely monitoring the cardiac health of COVID-19 survivors.

**Objective:**

This study aims to test a virtual, in-home healthcare monitoring model of care for detection of clinical symptoms and impacts on COVID-19 survivors. It also aims to demonstrate system usability and feasibility.

**Methods:**

This open label, prospective, descriptive study was conducted in South Western Sydney. Included in the study were patients admitted to the hospital with the diagnosis of COVID-19 between June 2021 and November 2021. Eligible participants after consent were provided with a pulse oximeter to measure oxygen saturation and a S-Patch EX to monitor their electrocardiogram (ECG) for a duration of 3 months. Data was transmitted in real-time to a mobile phone *via* Bluetooth technology and results were sent to the study team *via* a cloud-based platform. All the data was reviewed in a timely manner by the investigator team, for post COVID-19 related symptoms, such as reduction in oxygen saturation and arrhythmia.

**Outcome measure:**

This study was designed for feasibility in real clinical setting implementation, enabling the study team to develop and utilise a virtual, in-home healthcare monitoring model of care to detect post COVID-19 clinical symptoms and impacts on COVID-19 survivors.

**Results:**

During the study period, 23 patients provided consent for participation. Out of which 19 patients commenced monitoring. Sixteen patients with 81 (73.6%) valid tests were included in the analysis and amongst them seven patients were detected by artificial intelligence to have cardiac arrhythmias but not clinically symptomatic. The patients with arrhythmias had a higher occurrence of supraventricular ectopy, and most of them took at least 2 tests before detection. Notably, patients with arrhythmia had significantly more tests than those without [*t*-test, *t* (13) = 2.29, *p *< 0.05].

**Conclusions:**

Preliminary observations have identified cardiac arrhythmias on prolonged cardiac monitoring in 7 out of the first 16 participants who completed their 3 months follow-up. This has allowed early escalation to their treating doctors for further investigations and early interventions.

## Introduction

Coronavirus disease (COVID-19) is an infectious disease caused by the SARS-CoV-2 virus. Some people may experience a wide range of ongoing health problems post COVID-19 infection which may last for weeks, months or years (1, 2). These health problems have been collectively termed as post-COVID conditions or long COVID (3, 4). The severity of symptoms vary between different individuals which can range from very mild to severe. The most commonly reported long COVID symptoms are fatigue, dyspnoea, depression, anxiety, memory loss, sleep disorder and concentration difficulties (5). The follow-up studies have reported that the symptoms could persist in patients up to 2 years or more after acute infection (6, 7). However the occurrence and duration of long COVID is still unclear due to heterogeneity in the studies with respect to follow-up duration, case definitions, study cohort, assessment tools and study design (6–9).

One of the common long term health issues faced by COVID-19 survivors is post-exertional malaise (PEM), a worsening of symptoms and reduction in function after physical or mental activity (10). In some cases, the symptoms are fairly constant, while others may experience fluctuation in their symptoms (11). Multiple types of triggers can lead to an extremal deterioration of the overall health condition (12). In an international online survey of 3,762 individuals with suspected or confirmed COVID-19 illness, more than 85% of individuals experienced relapses of their symptoms, with exercise, physical or mental activity, and stress being the main triggers (8). Vital signs such as heart rate, blood pressure, and respiratory rate are the most sensitive to the PEM triggers and thus measuring and monitoring these vitals could help identify and prevent any further deterioration in patient's condition (12–14).

Sensors and commercial wearable devices have been widely used to monitor vital signs in COVID-19 patients over long periods of time (15–19). Studies utilizing data from fitbit devices have shown modification in the Resting Heart Rate (RHR) for up to 3 months following symptom onset with substantial intraindividual variability. A pattern of bradycardia and tachycardia in the RHR were also observed in the patients over this period (17, 18). For patients with underlying heart conditions and respiratory disease, COVID-19 can have fatal consequences. There is increasing evidence that COVID-19 survivors are at increased risk of experiencing a wide range of cardiovascular complications regardless of age, race, sex, symptom severity, underlying chronic condition and care setting (20–24). Recent studies have shown a significant variation in parasympathetic modulation and Heart Rate Variability (HRV) in patients post COVID-19 infection (25–27), thus highlighting the need for early identification of patients at risk of clinical deterioration.

CardioVascular Disease (CVD) is one of our biggest health problems, killing an Australian every 12 min (28). Cardiac arrhythmias in particular, Atrial Fibrillation (AF) increase risk of stroke and heart failure, and are a common cause of death (29). COVID-19 can cause myocardial (heart muscle) damage and cardiac arrhythmias, increasing the risk of stroke and heart failure (30–32). Up to 30% of patients hospitalised with COVID-19 in 2019 had evidence of myocardial involvement and acute cardiac injury was associated with higher morbidity and mortality (21, 30). An in-depth analysis of federal health data indicated that the risk and 1-year burden of CVD in survivors of acute COVID-19 are substantial (23).

Most people with COVID-19 do not require admission to hospital as they are able to recover at home. With a disease as infectious as COVID-19, remaining in isolation plays a key role in preventing transmission however it is also necessary to ensure patients are receiving the care they need. Virtual, in-home digital healthcare programs for confirmed COVID-19 cases have been implemented globally and in Australia in response to the pandemic, however, more evidence is needed on their adaptation and implementation. There are no validated models or clear guidelines for remotely monitoring the cardiac health of COVID-19 survivors. Despite reasonably effective vaccines, COVID-19 continues to be a serious public health problem. The significant negative economic impact has driven government policy decisions to remove mask mandates and lift most COVID-19 restrictions. However, with the emergence of new variants and increasing surges in case numbers, the ultimate health impact is huge. Furthermore, there is no definite understanding of the chronic impacts of COVID-19 on heart, lungs, organs and brain. This warrants the development of innovative and alternate approaches to designing collaborative models of care. A validated model for digital health solution that detects cardiac health deterioration early and reduces the burden on hospital-based clinical care will benefit patients, primary and secondary health carers, and acute-care hospitals. Therefore, early screening of convalescing patients *via* a virtual, in-home healthcare program may reduce the population burden of long-term CVD from COVID-19.

## Materials and methods

### Study aim

This study aimed to demonstrate usability and feasibility of the implementation of an innovative, novel model of digital healthcare in the primary healthcare setting for vulnerable populations affected by COVID-19 survivors. We had combined two smart, wearable/portable biosensor technologies to remotely identify clinical signs and symptoms of CVD in patients who had returned home after hospitalisation due to COVID-19 infection.

### Study design and intervention

This was an open label, prospective, descriptive study of a custom-designed virtual, in-home healthcare monitoring model of care to detect ongoing clinical symptom and impacts on COVID-19 survivors. The model of care utilised bio-instrumentation technologies, including pulse oximeter to measure oxygen saturation, and S-Patch EX to monitor the electrocardiogram (ECG) *via* mobile phone application which allowed clinicians to review the results *via* a cloud-based platform. These devices were approved for clinical use worldwide and have approval from the Therapeutic Goods Authority (TGA).

#### iHealth pulse oximeter

**Figure 1:**
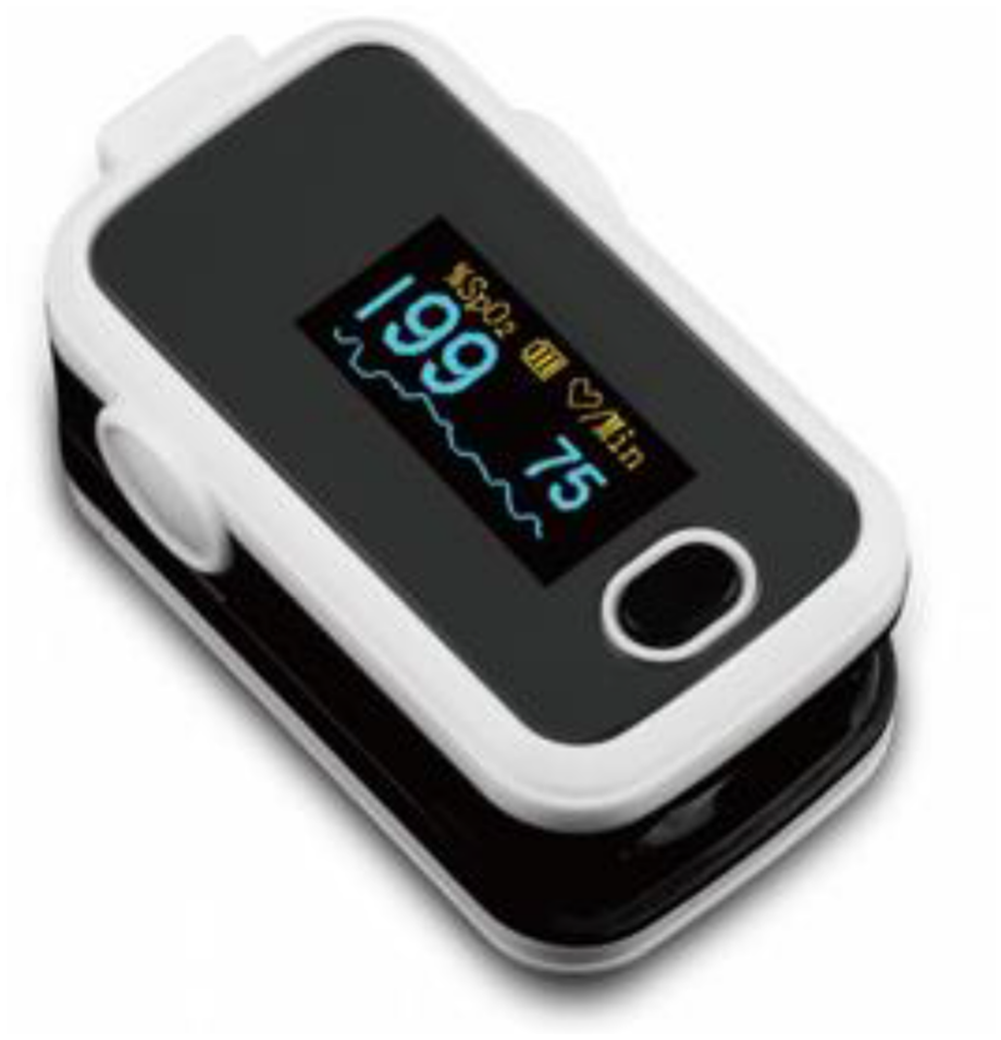
Ihealth pulse oximeter.

The iHealth wireless pulse oximeter worked by emitting beams of light through the finger and determining the blood oxygen levels based on light absorption (Figure 1). The participants were required to download the iHealth app on their smart phones which connected to their pulse oximeter to record their oxygen saturations, a minimum of 3 times a day.

#### S-Patch EX

**Figure 2:**
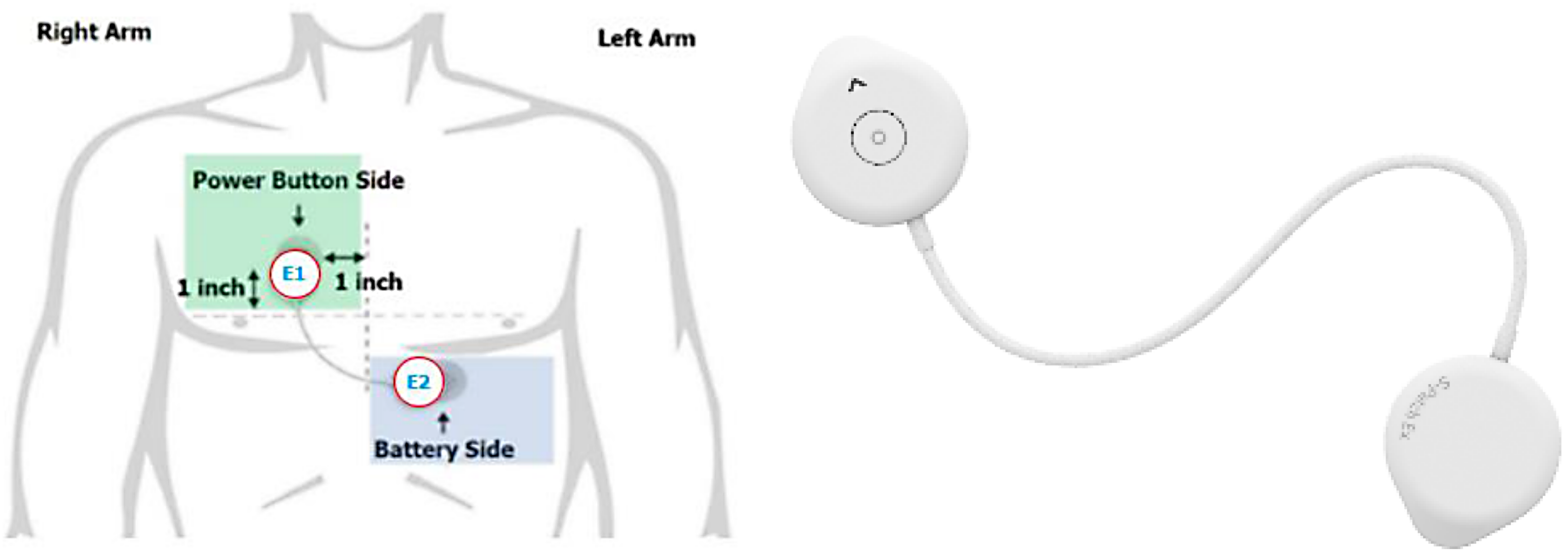
S-Patch Ex.

Wellysis, a Samsung SDS spinoff company, had developed a bio-processor; a single, compact chip that can measure Galvanic Skin Response (GSR) and PhotoPlethysmoGram (PPG) to sense pulsatile skin blood flow, ECG skin temperature, and body fat. This bio-processor is built into a device called the “S-Patch”, attachable to the chest with two electrode stickers (Figure 2); similar to a ward telemetry monitor that attaches to the chest *via* five electrode stickers. The bio-processor chip collected the data through the S-Patch device, which was transmitted in real-time to a mobile phone *via* Bluetooth technology. The participants were required to downloaded S Patch EX app onto their smartphones. The data was transmitted to the Cloud (e-server) for initial analysis and reporting, with this data being accessible with secure logon from desktops, tablets or other mobile devices. Our previous study had demonstrated the potential for the S-Patch wearable technology to be a clinically equivalent and improved solution for screening and diagnosis of arrhythmias whilst transforming into a more integrated and innovative model of care (33).

### Study setting

This study was conducted in South Western Sydney.

### Participants

Since this was a feasibility study, the sample for this study consisted of patients enrolled over the specific time period. All the patients admitted to the hospitals in the South Western Sydney region during June 2021 to November 2021 with the diagnosis of COVID-19 were screened for eligibility. Individuals were suitable for inclusion if they were 18 years of age or above, had a smartphone with Wi-Fi Internet at home and provided informed consent. Excluded from the study were patients who were pacemaker dependent or had Implantable Cardiac Defibrillator (ICD) and did not provide consent.

### Patient and public involvement

Patients or the public were not involved in the design, conduct, reporting or dissemination plans of this research. A survey of feedback from the patients was planned for electronic collection at 1, 2 and 3 months. There were five responses including one incomplete response and no conclusive results could be drawn. A number of healthcare worker representatives were involved in the development of the research question and the research methodology. De-identified preliminary study results were presented to key healthcare workers.

### Data collection

The list of admitted patients with COVID-19 diagnosis was obtained from the South Western Sydney Local Health District Emergency Operations Centre. All patients were screened and assessed for suitability against the inclusion and exclusion criteria. The identified eligible participants were called as per the telephone script. Interested participants were sent the Participant Information Sheet (PIS) and Consent Form (CF). Depending on the participants preferences the PISCF was sent electronically *via* the Veeva myconsent platform, email or post. Participants were given ample time to consider their participation in the study prior to signing of the consent form. They could also discuss the study with their doctor or family members before consenting. Participation in this study was strictly voluntary and all participants were required to provide informed consent prior to any study procedure. Follow up phone call will be made to the participant to answer any questions or if any escalation of their remote monitoring data. Participants have access to the phone number of the research team and are encouraged to make the calls if required.

Once the patients consented, they were provided with both the iHealth pulse oximeter and the S-Patch EX for up to 3 months before returning to the research team *via* a pre-paid envelope. Other consumables such as batteries and ECG dots required for S-Patch were also provided. No additional visit to hospital was required. The participant was then fitted with both the devices according to the Product Operations Manual, guided by an experienced nurse/researcher *via* teleconsultation. Data was transmitted in real-time to their mobile phone *via* Bluetooth technology and results were uploaded on a cloud-based platform accessible to the study team.

The data was reviewed daily by a study investigator (clinician) with any findings requiring escalations highlighted and forwarded to the research team's medical officers for further review and advice. Medical officers in the research team had access to the telemetry database to allow for real-time review of ECG strips. Based on the advice of the medical officer's telemetry review, the patient and their doctor were informed of the findings and recommendation of any interventions if required. These interventions included but were not limited to the following:

• If the participant was determined at risk for an adverse event such as AF, this information was shared with their doctor immediately, *via* phone call +/− fax of summary telemetry.
• If the patient was found to be experiencing a medical emergency, they were advised to present to the nearest hospital or call the ambulance for assistance.
• If no further action was required outside of ongoing monitoring, the summary was provided to the patient and their doctor for their records.
• Should the patients require specialist review, their local treating doctor was expected to organise specialist consultations.

### Analysis

The data analysis was conducted with Pandas 1.3.5 (34), an open-source data analysis tool. The statistical test including *t*-test was conducted with Scipy 1.7.3 (35), an open-source software for mathematics, science, and engineering. The analysis results were visualized using Maplotlib 3.5.1 (36).

## Results

During the study period, 23 patients provided consent for participation. Out of which four were considered non-compliant as they did not commence any monitoring. The 19 patients who commenced monitoring had a mean age of 49 years (SD = 12.27), 57.9% (*n* = 11) of them were female.

### S-Patch EX

The data analysis for the ECG *via* the S-Patch Ex included 16 patients with 81 (73.6%) valid tests out of 19 patients (total of 110 tests). Amongst 29 tests, 13 were excluded due to lack of valid data (*N* = 13) and other 16 were ruled out as they were less than 30 min (*N* = 16). Seven patients were detected by the Artificial Intelligence (AI) algorithms from the S-patch EX monitoring system, to have cardiac arrhythmias, including Atrial Fibrillation (AF), SupraVentricular Tachycardia (SVT), and Ventricular Tachycardia (VT).

#### Data summary

Out of those 16 patients with 81 valid tests (16 days of data collected on average) study reported, 7 patients had cardiac arrhythmias, including AF, SVT, and VT (Patient 3, 6, 7, 8, 10, 12, 18).

**Figure 3:**
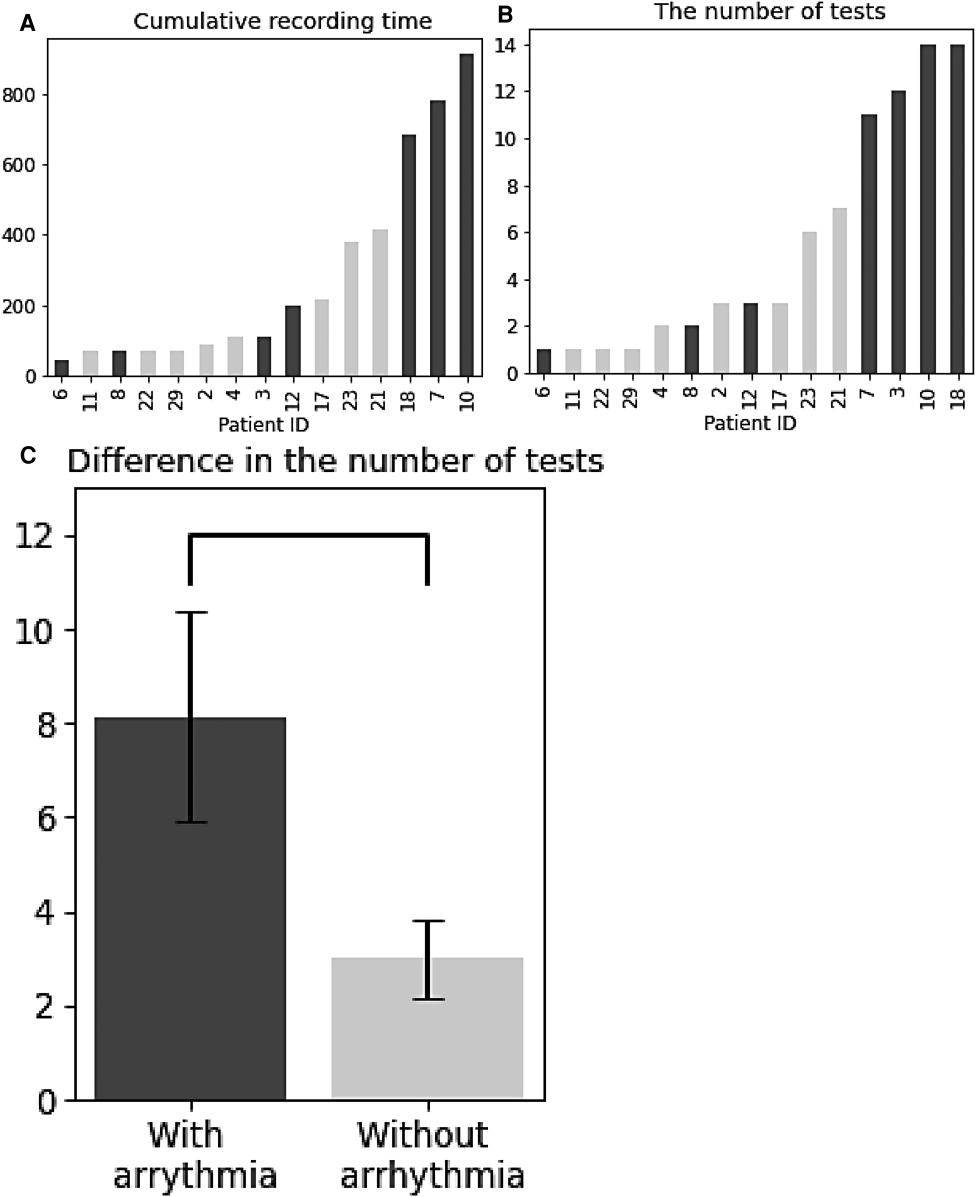
Overall data summary. (**A**) Cumulative recording time for patients. (**B**) The number of tests for patients. (**C**) Difference in the number of tests between patients with and without arrythmia. (**A–C**) Patients with arrhythmia are shown in dark colour.

Figures 3A,B shows the cumulative recording time of all the patients and the number of tests, and seven patients with arrhythmia were shown in dark colour. Most of the patients with arrhythmia took at least 2 tests before detection, except for Patient 6, who had abnormal rhythms in the first test. Notably, patients with arrhythmias had significantly more tests than those without [*t*-test, *t* (13) = 2.29, *p *< 0.05, Figure 3C].

#### Mean of QRS complexes, SVE%, and VE% for all patients

**Figure 4:**
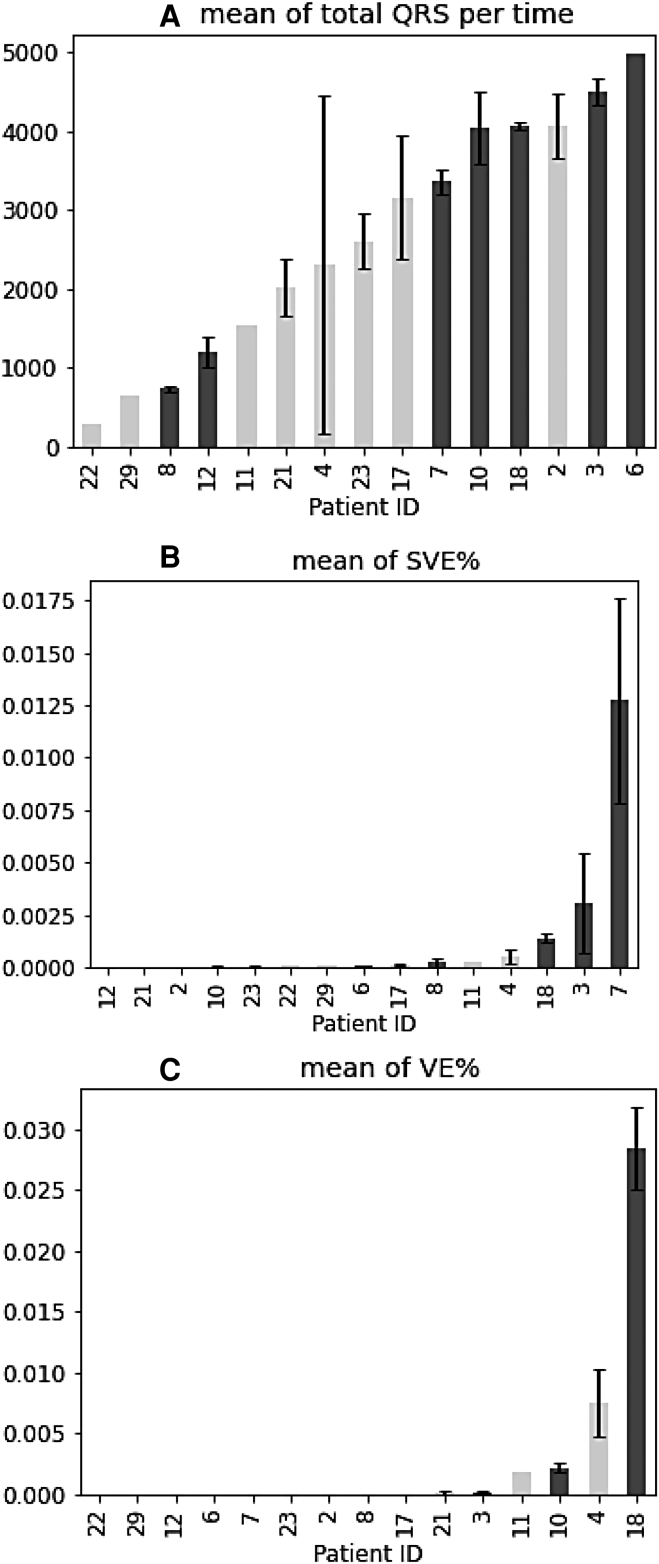
Mean of QRS complexes, SVE%, and VE% for all patients. (**A–C**) Patients with arrhythmia are shown in blue. Error bars indicate ± standard error of the mean.

The percentage (%) of total QRS in relation to the total recording time per patient was analysed (Figure 4A) and it did not show any trend between the number of QRS complexes and cardiac arrhythmias occurrence. The average of SVE% (the ratio of SVE to the number of QRS) by patients was observed and the patients with arrhythmias had a higher occurrence of SVE on the tests they performed (Figure 4B). The means of SVE% for the patients (especially Patient 18, 3, 7) tended to be higher than for the other patients (Figure 4B). In summary, the % occurrence of SVE per test might be correlated to cardiac arrhythmias. In contrast to SVE, the average of VE% (the ratio of VE to the number of QRS) was not correlated to cardiac arrhythmias since no trend was observed (Figure 4C).

#### Change of QRS complexes, SVE%, and VE% across tests within arrhythmia patients

**Figure 5:**
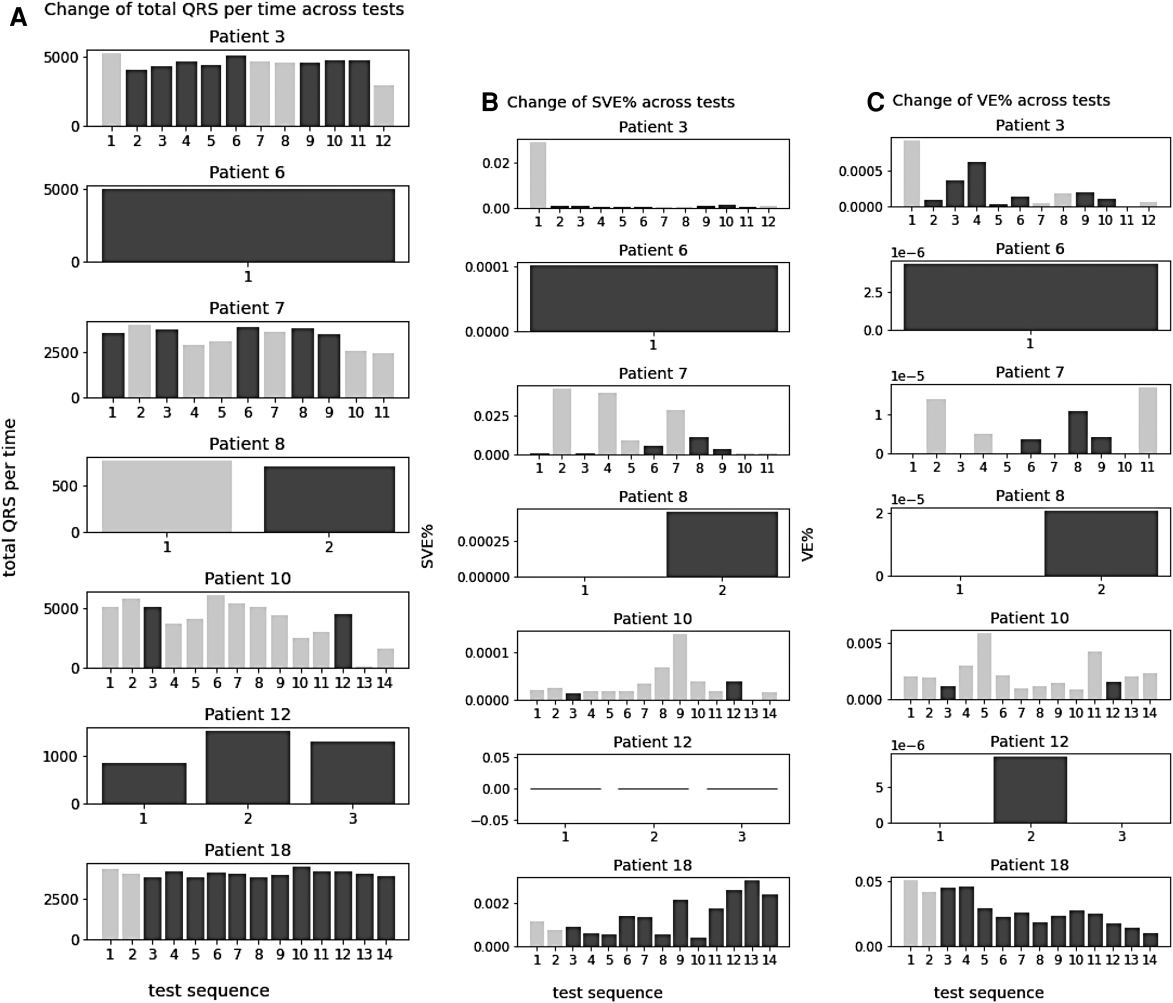
Change of QRS complexes, SVE%, and VE% across tests within arrhythmia patients. (**A–C**) Tests with arrythmia detected are shown in blue.

After examining overall trend across all patients and investigating the change of these statistics across tests within the patients with cardiac arrhythmias (Patient 3, 6, 7, 8, 10, 12, 18). There was no trend between the number of QRS complexes and arrhythmias occurrence throughout the tests. They did not increase or decrease across the tests, and the test with arrhythmias did not show particularly high or low number of QRS (Figure 5-1).

The change of SVE % (the ratio of SVE to the number of QRS) and VE % (the ratio of VE to the number of QRS) was also analysed and no trend was found in relation to the arrhythmias across tests. They did not increase or decrease across the tests, and the test with arrhythmias did not show particularly high or low SVE% and VE%.

### iHealth pulse oximeter

Analysis of the data from the iHealth pulse oximeter was incomplete due to the lack of consistent and/or valid data as required for the study. Participants were required to download the app and take pulse oximeter readings a minimum of three times a day and send the data *via* the app. While most of the participants were focused on the S-Patch EX and its app, they failed to record and send their oxygen saturations despite of reminder and/or compliant with the frequency and the reporting. Participants may have recorded their data at various times during the study however this could not be determined as there was limited iHealth data (*n* = 2) received by the study team. Incidentally, the S-Patch EX also had the capability to collect heart rate data. All the readings collected and submitted were within the normal range.

### Refinement of the protocol and communication platform

The study team met weekly to evaluate and refine the protocol, Standard Operation Procedure (SOP) process of care pathway and communication platform, for the proposed larger study. The processes of care pathway included (i) patients' selection to determine eligibility, (ii) onboarding of patients to the remote in-home monitoring service (provision of information to patient and/or carer on monitoring process), (iii) monitoring (including recording of observations, communication of the information, assessment of the information by the project and medical team), (iv) escalation (if required), and (v) discharge from the pathway.

The results of the experience and feedback of this preliminary group of participants provided valuable information for the study protocol amendments, which were submitted and approval by the Human Research Ethics Committee for further refinement. Some of the key amendments included:

• Instead of using the Bluetooth connection for the iHealth app to record their pulse oximetry readings, future participants will record their readings into the S-Patch Ex app. This information will then be uploaded with the S-Patch EX information automatically.
• Instead of requirement for 24/7 monitoring for a period of up to 3 months, those participants who were doing reasonably well and were unable to monitor every day, an option was given to monitor during weekends OR 2–3 consecutive days per week OR only during their “bad days” when they experience any symptom (extreme fatigue, shortness of breath, heart palpitations, chest pain or tightness problems with memory and concentration changes to taste and smell joint and muscle pain).

## Discussion

Various remote home monitoring models have been designed and implemented for COVID-19 patients who were stable enough to be monitored at home providing early detection of deterioration, manage care escalation, avoid unwarranted hospital visit and further reduce strain on the hospitals (14, 37). Most of the models mainly focused on monitoring patients during their infectious period. Our study aimed to co-design and implement an innovative, novel model of virtual, in-home healthcare monitoring using advanced technologies and AI for vulnerable populations affected by long-term COVID symptoms and assessing its impacts on COVID-19 survivors. The project team had also developed and utilised a communications platform to connect all the healthcare professionals in the cardiac care pathway to ensure the patients received early screening *via* virtual, in-home healthcare program and activate appropriate interventions if required. This study identified recruitment barriers and developed guided strategies to overcome these so as to determine the most feasible and usable monitoring protocol.

Arrhythmias, such as AF are associated with an increased risk of stroke and heart failure. Recent studies (23, 24) have shown increased risk of arrhythmia related disorders among COVID survivors. In our study, the continuous data from S-Patch EX and its AI technology has enabled further investigation on the changes of cardiac physiology for the study participants. In particular, it showed that patients detected to have arrhythmias were tested significantly more than those who were not. Although it should be interpreted with caution whether more tests led to more detection of cardia arrhythmia or whether more tests were performed in patients with discomfort, this result suggested that a sufficient number of tests might help to detect cardiac arrhythmias. The patients with arrhythmias had a higher occurrence of SVE, while the number of QRS complexes and VE did not seem relevant to the abnormal heart rhythms. With the change of these statistics across tests within patients, no trend in SVE was observed. According to Figure 4B, patients with arrhythmias showed higher SVE% than other patients, but individual tests did not show any trend in relation to arrhythmias. This might indicate that although the average number of SVEs is important, the change over time or the frequency at the time of arrhythmias onset could not be significant.

### Potential implications for policy and communications

An important finding from this study is the co-design and deployment of an innovative virtual care to fill the current gap, leveraging advancements in technologies to expedite the development and implementation of a digital intelligence model of cardiac health screening and monitoring. It has also improved patients' access to early screening and facilitation of early diagnostic and intervention from their primary healthcare partners.

### Limitations of study

There are a number of limitations of this study. The limitation of available and approved technologies in Australia was a barrier to optimise the co-design and option for the solution. The inability to integrate both the S-Patch Ex and the iHealth pulse oximeter into a single app was a barrier which gave rise to technical challenges and issues with compliance resulting in lack of consistent and valid data from the pulse oximeter. The full function of the technologies had not been utilised, for example, the S-Patch EX devices have some benefits suitable for long-term and patient-engaged test processes. Patients can conveniently wear the S-Patch EX on their bodies themselves, so it was possible to start tests easily when patients feel symptoms as in the study. Patients also can remotely test their ECG at home by directly transmitting the ECG data to medical staff without frequent hospital visits.

While this study has demonstrated feasibility for the solution in a real clinical setting and further adaptation is currently in progress, a well-designed prospective study with larger sample will be required. A non-randomised interventional study has been designed to further evaluate this virtual healthcare solution. This has the potential to make the in-home service more accessible, affordable and effective to boost patients' satisfaction with our healthcare system and reduce the burden of healthcare through efficiency gains.

## Conclusion

Implementation of the S-Patch device is anticipated to result in a reduction of healthcare costs through more efficient medical monitoring/data collection *via* the utilisation of new technology; as well as being able to improve patient outcomes by encouraging closer patient and health professional relationships through collaborative data monitoring and open discussion approaches.

## Data Availability

The raw data supporting the conclusions of this article will be made available by the authors, without undue reservation.
